# NBA-Palm: prediction of palmitoylation site implemented in Naïve Bayes algorithm

**DOI:** 10.1186/1471-2105-7-458

**Published:** 2006-10-17

**Authors:** Yu Xue, Hu Chen, Changjiang Jin, Zhirong Sun, Xuebiao Yao

**Affiliations:** 1 Laboratory of Cellular Dynamics, Hefei National Laboratory for Physical Sciences, and the University of Science and Technology of China, Hefei, China 230027; 2 Institute of Bioinformatics and Systems Biology, MOE Key Laboratory of Bioinformatics, State Key Laboratory of Biomembrane and Membrane Biotechnology, Department of Biological Sciences and Biotechnology, Tsinghua University, Beijing, China 100084; 3 Department of Physiology and Cancer Research Program, Morehouse School of Medicine, Atlanta, GA 30310, USA

## Abstract

**Background:**

Protein palmitoylation, an essential and reversible post-translational modification (PTM), has been implicated in cellular dynamics and plasticity. Although numerous experimental studies have been performed to explore the molecular mechanisms underlying palmitoylation processes, the intrinsic feature of substrate specificity has remained elusive. Thus, computational approaches for palmitoylation prediction are much desirable for further experimental design.

**Results:**

In this work, we present NBA-Palm, a novel computational method based on Naïve Bayes algorithm for prediction of palmitoylation site. The training data is curated from scientific literature (PubMed) and includes 245 palmitoylated sites from 105 distinct proteins after redundancy elimination. The proper window length for a potential palmitoylated peptide is optimized as six. To evaluate the prediction performance of NBA-Palm, *3*-fold cross-validation, *8*-fold cross-validation and Jack-Knife validation have been carried out. Prediction accuracies reach 85.79% for *3*-fold cross-validation, 86.72% for *8*-fold cross-validation and 86.74% for Jack-Knife validation. Two more algorithms, RBF network and support vector machine (SVM), also have been employed and compared with NBA-Palm.

**Conclusion:**

Taken together, our analyses demonstrate that NBA-Palm is a useful computational program that provides insights for further experimentation. The accuracy of NBA-Palm is comparable with our previously described tool CSS-Palm. The NBA-Palm is freely accessible from: .

## Background

Protein palmitoylation is a reversible lipid modification that plays important roles in cell signaling associated with cellular dynamics and plasticity. However, very little is known about the molecular mechanism underlying this modification and regulation in cells. Palmitoylation, also known as S-acylation, is one of the most ubiquitous post-translational modifications (PTM), reversibly attaching a 16-carbon saturated fatty acid as lipid palmitate (C16:0) to cysteine residues in protein substrates through thioester linkage [1-6]. Biochemically, palmitoylation increases the hydrophobicity of proteins to promote protein-membrane association [1-6]. Also, palmitoylation modifies numerous proteins to control protein-protein interaction [7-9], intracellular trafficking [10,11], lipid raft targeting [12,13], and proteins' activities [8,14], etc. Moreover, palmitoylation has been implicated in a variety of biological and physiological processes, including signal transduction [14,15], mitosis [16], neuronal development [3,6], and apoptosis [17], etc. Although protein palmitoylation has attracted extensive attention, its molecular mechanisms still remain to be elusive.

Identification of palmitoylation sites is essential for a better understanding the molecular regulation of palmitoylation process. To date, only a few palmitoylation sites have been experimentally identified. Although several efficient techniques, such as mass spectrometry (MS), have been employed recently, most of the known palmitoylation sites are mapped by mutagenesis of candidate cysteine residues with conventional biochemical methods. The features of substrate specificity for palmitoylation is still unclear and most previous studies have proposed that there is no common and canonical consensus sequence/motif for palmitoylation [1,3-5].

Moreover, only a few palmitoyltransferases have been identified although palmitoylation of proteins has been known for many years [2,4,18,19]. Palmitoylation of proteins can be carried out in both enzyme- and nonenzyme-dependent manners [5,18-20]. These intrinsic but diversified characteristics of palmitoylation introduce great difficulties into choosing appropriate candidate cysteine residues in the substrates for further experimental manipulation. Thus, *in silico *prediction of palmitoylation sites implemented in an apt algorithm/approach is in urgent need and insightful for further experimental design.

Previously, we developed a computational program named CSS-Palm, deployed with the approach of Clustering and Scoring strategy [21]. In that work, the data set for training was curated from scientific literature (PubMed) with 210 experimentally verified palmitoylated sites from 83 distinct proteins (referred to as old data set). Due to the fast pace of research progress in this area, more palmitoylation sites have been identified since our last publication of CSS-Palm. After survey recent progress and redundancy elimination, the final data set includes 245 non-homologous sites from 105 proteins (referred to as new data set, see in Table 1). We then employ several machine learning algorithms including Naïve Bayes [22], Support Vector Machines (SVMs) [23] and RBF Networks [24] for palmitoylation site prediction. Also, the proper window length for a potential palmitoylated peptide has been optimized. The accuracy of prediction performance fluctuates from 82% to 86%. By comparison, the Naïve Bayes approach achieves the best accuracy of 85.79% for *3*-fold cross-validation, 86.72% for *8*-fold cross-validation and 86.74% for Jack-Knife validation, with the window length of six. Thus, we construct a computational web service of NBA-Palm – prediction of palmitoylation site implemented in Naïve Bayes algorithm. And the prediction performance is comparable with our previous work of CSS-Palm.

**Table 1:** The detailed description of data set.

Data set	Old	New	
		original	Clear redundant
protein	84	111	105
sites	209	266	245
non-sites	720	1017	977

## Results & discussions

### Functional analysis of Palmitoylated Proteins

In order to elucidate the molecular determinants responsible for protein palmitoylation, we downloaded the GO annotation files for Uniprot from EBI-GOA [25] for processing. In our non-redundant data set with 105 palmitoylated proteins, we have observed 455 distinct GO categories. Table 2 shows the top five Gene Ontology (GO) entries of biological processes, molecular functions and cellular components of palmitoylated proteins.

**Table 2:** Top five Gene Ontology (GO) groups of biological processes, molecular functions and cellular components in palmitoylated proteins.

**GO Symbol**	**Name of Gene Ontology**	**No. of Proteins**
*Top five biological process*		
GO:0007165	signal transduction	26
GO:0007186	G-protein coupled receptor protein signaling pathway	21
GO:0006810	transport	16
GO:0006811	ion transport	7
GO:0007155	cell adhesion	7
		
*Top five molecular function*		
GO:0005515	protein binding	41
GO:0004872	receptor activity	27
GO:0004871	signal transducer activity	25
GO:0004930	G-protein coupled receptor activity	15
GO:0001584	rhodopsin-like receptor activity	14
		
*Top five cellular component*		
GO:0016020	membrane	70
GO:0016021	integral to membrane	54
GO:0005886	plasma membrane	24
GO:0005887	integral to plasma membrane	19
GO:0005783	endoplasmic reticulum	9

The most abundant GO item of biological process in which palmitoylated proteins are implicated is "signal transduction" (26 proteins). The other four biological processes are "G-protein coupled receptor protein signaling pathway" (21 proteins), "transport" (16 proteins), "ion transport" (7 proteins) and "cell adhesion" (7 proteins). The most enriched GO group of molecular function is "protein binding" (41 proteins), while the other four highly-abundant molecular functions are "receptor activity" (27 proteins), "signal transducer activity" (25 proteins), "G-protein coupled receptor activity" (15 proteins) and "rhodopsin-like receptor activity" (14 proteins). Again, the most frequent GO entry of cellular component is "membrane" (70 proteins), and the other four highly-frequent cellular components are "integral to membrane" (54 proteins), "plasma membrane" (245 proteins), "integral to plasma membrane" (19 proteins) and "endoplasmic reticulum" (9 proteins).

Taken together, the computational analyses of the palmitoylated proteins support the notion that palmitoylated proteins carry diversified cellular functions. The result points to two conclusions. First, the data set is general enough and suitable for our prediction work as training data set. Second, computational tools which can accelerate palmitoylation function research are valuable and helpful.

### Performance of NBA-Palm

**Figure 1:**
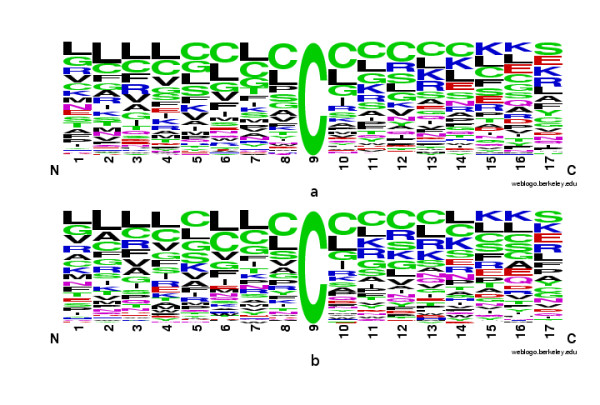
The sequence logos of palmitoylation sites. Both two logos show that around palmitoylation sites there is a Leucine/Cysteine-rich region. A taller letter indicates that this kind of residue is more frequently used. (a) on old data set; (b) on new data set.

We carried out *3*-fold cross-validation, *8*-fold cross validation and the Jack-Knife validation to evaluate the performance of NBA-Palm (shown in Table 3 and Table 4). On the old data set, NBA-Palm achieves best average MCC of 0.594 with window length of six. On the new data set, the best average MCC is 0.548 with the same optimized window length of six. The prediction performances on the old and new data set are very similar. However, the performance on the new data set is slightly lower than that on the old data set. To find out the reason of this performance decrease, we built sequence logos [26] on the old and new data sets (shown in Figure 1a and Figure 1b). Both two logos show that around palmitoylation sites there is a Leucine/Cysteine-rich region. Comparison of the two logos leads to the observation that the pattern of the old data set is slightly stronger than that of the new data set. This may explain why performance of NBA-Palm on the new data set is slightly lower.

**Table 3:** Comparison of the prediction performance for three machine learning algorithms on old data set.

**Algorithm**	**Window length**	**3-fold cross-validation**				**8-fold cross-validation**				**Jack-Knife validation**				**Average MCC**	**Max MCC difference**
		*Ac (%)*	*Sn (%)*	*Sp (%)*	*MCC*	*Ac (%)*	*Sn (%)*	*Sp (%)*	*MCC*	*Ac (%)*	*Sn (%)*	*Sp (%)*	*MCC*		
**Naïve Bayes^1^**	3	85.25%	54.39%	94.21%	0.5438	86.01%	55.98%	94.72%	0.5679	86.33%	57.42%	94.72%	0.5795	0.5637	0.0357
	4	85.97%	56.46%	94.54%	0.5677	86.29%	56.94%	94.81%	0.5776	86.44%	58.85%	94.44%	0.5851	0.5768	0.0174
	5	85.86%	58.53%	93.80%	0.569	86.11%	58.53%	94.12%	0.5757	86.22%	58.37%	94.31%	0.5783	0.5743	0.0093
	**6**	**85.93%**	**60.13%**	**93.43%**	**0.5744**	**86.58%**	**62.68%**	**93.52%**	**0.5967**	**86.98%**	**64.11%**	**93.61%**	**0.6099**	**0.5937**	**0.0355**
	7	86.15%	60.61%	93.56%	0.5811	86.19%	60.61%	93.61%	0.5820	86.44%	61.72%	93.61%	0.5909	0.5847	0.0098
	8	86.01%	60.29%	93.47%	0.5766	86.08%	60.13%	93.61%	0.5781	86.01%	62.20%	92.92%	0.5811	0.5786	0.0045
**RBF Network^2^**	3	84.39%	49.92%	94.40%	0.5104	85.15%	53.91%	94.21%	0.5399	83.85%	50.72%	93.47%	0.4975	0.5159	0.0424
	4	85.11%	53.59%	94.26%	0.5382	85.58%	55.18%	94.40%	0.5543	86.11%	57.89%	94.31%	0.5745	0.5557	0.0363
	5	85.97%	57.10%	94.35%	0.569	85.61%	57.26%	93.84%	0.5596	85.36%	57.42%	93.47%	0.5534	0.5607	0.0156
	6	86.04%	57.42%	94.35%	0.5716	86.11%	59.01%	93.98%	0.5767	85.79%	59.33%	93.47%	0.5689	0.5724	0.0078
	7	85.36%	58.53%	93.15%	0.556	85.65%	57.89%	93.70%	0.5620	86.65%	58.85%	94.72%	0.591	0.5697	0.0350
	8	85.07%	57.26%	93.15%	0.5456	85.43%	57.10%	93.66%	0.5545	86.76%	58.85%	94.86%	0.594	0.5647	0.0484
**SVM^3^**	3	84.46%	56.62%	92.55%	0.5285	85.15%	56.14%	93.56%	0.5448	86.44%	59.81%	94.17%	0.5869	0.5534	0.0584
	4	83.82%	59.97%	90.74%	0.5229	84.03%	58.37%	91.48%	0.5231	82.24%	52.15%	90.97%	0.4616	0.5025	0.0615
	5	82.99%	59.81%	89.72%	0.5041	83.24%	59.97%	90.00%	0.5100	80.52%	53.11%	88.47%	0.4272	0.4804	0.0828
	6	83.10%	62.52%	89.07%	0.5156	83.75%	63.48%	89.63%	0.5326	82.13%	60.77%	88.33%	0.4893	0.5125	0.0433
	7	81.45%	61.72%	87.18%	0.4793	83.21%	63.48%	88.94%	0.5212	83.53%	63.16%	89.44%	0.5269	0.5091	0.0476
	8	80.95%	61.88%	86.48%	0.4702	82.10%	63.16%	87.59%	0.4974	82.45%	63.16%	88.06%	0.5046	0.4907	0.0344

1. Default parameters of WEKA software package were used.

2. Ridge value was set to 10E-8.

3. Polynomial kernel, exponent was set to 1, complexity value C to 0.1.

**Table 4:** Comparison of the prediction performance for three machine learning algorithms on new data set.

**Algorithm**	**Window length**	**3-fold cross-validation**				**8-fold cross-validation**				**Jack-Knife validation**				**Average MCC**	**Max MCC difference**
		*Ac (%)*	*Sn (%)*	*Sp (%)*	*MCC*	*Ac (%)*	*Sn (%)*	*Sp (%)*	*MCC*	*Ac (%)*	*Sn (%)*	*Sp (%)*	*MCC*		
**Naïve Bayes^1^**	3	84.64%	43.95%	94.85%	0.4629	85.08%	44.08%	95.36%	0.4767	85.27%	45.31%	95.29%	0.4857	0.4751	0.0228
	4	85.43%	49.52%	94.44%	0.5017	85.62%	51.29%	94.23%	0.512	85.76%	51.43%	94.37%	0.5162	0.51	0.0145
	5	85.49%	51.97%	93.89%	0.5101	86.17%	53.74%	94.30%	0.5339	86.58%	54.69%	94.58%	0.5479	0.5306	0.0378
	**6**	**85.79%**	**54.01%**	**93.76%**	**0.5241**	**86.72%**	**57.28%**	**94.10%**	**0.5582**	**86.74%**	**58.37%**	**93.86%**	**0.5618**	**0.548**	**0.0377**
	7	85.65%	54.42%	93.48%	0.5216	86.52%	56.87%	93.96%	0.5519	86.58%	57.14%	93.96%	0.5541	0.5425	0.0325
	8	85.79%	55.37%	93.42%	0.528	86.20%	57.55%	93.38%	0.545	86.25%	56.73%	93.65%	0.5442	0.5391	0.0170
**RBF Network^2^**	3	84.12%	45.17%	93.89%	0.4516	85.05%	45.44%	94.98%	0.4794	84.62%	46.12%	94.27%	0.4684	0.4665	0.0278
	4	84.83%	46.39%	94.47%	0.4755	85.57%	49.12%	94.71%	0.5046	85.68%	49.80%	94.68%	0.5095	0.4965	0.0340
	5	85.32%	48.98%	94.44%	0.4971	85.35%	48.57%	94.58%	0.4967	86.91%	54.29%	95.09%	0.5565	0.5168	0.0598
	6	85.38%	51.29%	93.93%	0.5051	85.76%	50.61%	94.58%	0.514	85.92%	50.20%	94.88%	0.5178	0.5123	0.0127
	7	85.08%	51.16%	93.59%	0.4965	86.33%	53.47%	94.58%	0.5379	86.99%	53.88%	95.29%	0.558	0.5308	0.0615
	8	85.43%	50.48%	94.20%	0.5043	86.42%	54.01%	94.54%	0.5416	87.15%	55.51%	95.09%	0.5664	0.5374	0.0621
**SVM^3^**	3	84.72%	48.44%	93.82%	0.4785	85.73%	48.57%	95.05%	0.5081	85.35%	47.35%	94.88%	0.4935	0.4934	0.0296
	4	82.84%	49.66%	91.16%	0.4349	84.89%	51.16%	93.35%	0.4914	84.78%	52.24%	92.94%	0.4919	0.4727	0.0570
	5	83.17%	52.65%	90.82%	0.4541	85.32%	55.24%	92.87%	0.5155	86.82%	60.41%	93.45%	0.5694	0.513	0.1153
	6	82.87%	52.79%	90.41%	0.4478	85.30%	57.55%	92.26%	0.5221	84.37%	57.55%	91.10%	0.4999	0.4899	0.0743
	7	81.53%	53.33%	88.60%	0.4213	84.70%	58.37%	91.30%	0.5104	88.46%	64.49%	94.47%	0.6234	0.5184	0.2021
	8	81.01%	53.33%	87.96%	0.4108	84.48%	60.54%	90.48%	0.5132	85.52%	61.22%	91.61%	0.5393	0.4878	0.1285

1. Default parameters of WEKA software package were used.

2. Ridge value was set to 10E-8.

3. Polynomial kernel, exponent was set to 1, complexity value C to 0.1.

### Comparison of Prediction Performance with several machine learning algorithms

Besides Naïve Bayes, we also adopt two additional machine learning algorithms, RBF networks and support vector machines (SVMs), to predict palmitoylation site. Table 3 and Table 4 show the detailed performances of the three algorithms on the old and new data sets, separately. Several conclusions can be reached: firstly, despite of its simple structure, Naïve Bayes is overall the best algorithm. However, its performance is only slightly better than that of the other two. Secondly, best window lengths for the three algorithms are not identical, e.g. on new data set 6 for Naïve Bayes, 8 for RBF networks and 7 for SVMs, according to average MCC of *3*-fold cross-validation, *8*-fold cross-validation and Jack-Knife validation. Thirdly, performances of Jack-Knife tests are often better than those of *3*-fold and *8*-fold cross-validations because there are more training data and less test data. Among the three algorithms, SVM has the largest differences in MCC between *3*-fold cross-validation, *8*-fold cross validation and Jack-Knife test while Naïve Bayes has the smallest. This implies that Naïve Bayes may be the most robust algorithm when changing the numbers of training data and test data. And the window length of Naïve Bayes algorithm is optimized as six by comparison of the average MCC. Hence, Naïve Bayes is a very simple-structured algorithm with high performance and robustness, which is extremely suitable for biological classification problems.

### Comparison with previously described analysis CSS-Palm

Performance comparison was carried out between NBA-Palm and the previously established method CSS-Palm [21] on the same old data set. Details are shown in Table 5. In the Jack-Knife validation, NBA-Palm performs comparatively with CSS-Palm in all metrics. However, in *3*-fold cross-validation, NBA-Palm achieves much higher MCCs, which is probably due to the volatility of the *3*-fold cross-validation, because the *3*-fold cross-validation uses less training data (2/3 of whole data set) and makes predictions on more testing data (1/3 of whole data set) while Jack-Knife validation uses all data but one for training. The result implies that the robustness of the Naïve Bayes method is probably inherited from the nature of probability theory. This is consistent with the conclusion above achieved in comparison of Naïve Bayes and SVM. In contrast, CSS-Palm is based on sequence/peptide homology scoring and clustering. And lacking a key sequence/peptide in training data might cause large changes in clustering results. Thus, CSS-Palm depends on training data heavily with less robustness.

**Table 5:** Comparison of prediction performances between NBA-Palm and CSS-Palm.

**Program**		**3-fold cross-validation**					**Jack-Knife validation**			
	Cut-off	*Ac (%)*	*Sn (%)*	*Sp (%)*	*MCC*	Cut-off	*Ac (%)*	*Sn (%)*	*Sp (%)*	*MCC*
**CSS-Palm**	4	43.34%	40.84%	100.00%	0.1667	4	87.00%	50.65%	97.62%	0.5946
	2.6	68.75%	67.78%	88.89%	0.2398	2.6	82.94%	82.16%	83.17%	0.5877
	1.5	88.70%	90.66%	44.44%	0.2247	1.5	56.67%	97.12%	44.86%	0.3672
**NBA-Palm**	0.869	86.61%	40.70%	100.00%	0.5891	0.745	86.66%	50.24%	97.23%	0.5809
	0.359	85.78%	67.90%	91.00%	0.5916	0.406	86.67%	67.46%	92.25%	0.6102
	0.011	60.78%	90.90%	52.00%	0.3630	0.016	56.41%	94.73%	45.28%	0.3475

* Cut-off values of NBA-Palm were chosen for convenience of performance comparison to CSS-Palm.

### Perspective of Future work

Our work points to several paths for further research. Firstly, as the proteomic techniques continue to be improved, more and more palmitoylation sites will be identified. We can expect that the accuracies will be further improved with more training data. Secondly, some other machine learning methods could be applied, i.e., decision trees [24] and hidden Markov models [27]. These approaches could be used separately or combined together to build potentially better models. Thirdly, evolutionary information, for example, phylogenetic conservation between human and mouse, can also be integrated into the prediction system to improve its accuracy.

## Conclusion

In this work, we present a new method for protein palmitoylation site prediction based on Naïve Bayes. The performance is satisfactorily high. Comparison between Naïve Bayes, RBF networks and SVMs was also carried out, and demonstrated that Naïve Bayes outperforms the other two methods. We also compared NBA-Palm with our previously established method CSS-Palm. The comparison demonstrates that NBA-Palm carries superior computing efficiency to CSS-Palm with equal predicting accuracy. These results indicate that Naïve Bayes is an effective classification algorithm for biological problems. In addition, with high specificity and sensitivity, NBA-Palm could be a valuable computational tool for functional proteomic biologists.

## Methods

### Data Preparation

Here we define the cysteine (C) residues that undergo palmitoylated modification as positive data (+), while those non-palmitoylated cysteine residues are regarded as negative data (-). Previously, we have collected 210 experimentally verified palmitoylation sites of 84 proteins [21]. Since palmitoylation-related research is updated rapidly, more and more palmitoylated sites have been identified and reported. We searched the PubMed with the keyword "palmitoylation" to collect new palmitoylation sites. Now the updated new data set contains 266 sites from 111 proteins (before March. 31^st^, 2006). We then retrieved the primary sequences of these proteins from Swiss-Prot/TrEMBL database [28]. The final curated data set is available upon request.

The positive data (+) set for training might contain several homologous sites from homologous proteins. If the training data are highly redundant with too many homologous sites, the prediction accuracy will be overestimated. To avoid the overestimation, we clustered the protein sequences from positive data (+) set with a threshold of 30% identity by BLASTCLUST [29], one program of clustering highly homologous sequences into distinct groups. If two proteins were similar with ≥30% identity, we re-aligned the proteins with BL2SEQ, a program in the BLAST package [29], and checked the results manually. If two palmitoylation sites from two homologous proteins were at the same position after sequence alignment, only one item was reserved while the other was discarded. Thus, we obtained a non-redundant positive data (+) of high quality with 245 palmitoylation sites from 105 proteins.

As previously described [30,31], the negative (-) sites were composed of non-annotated cysteine residues in the same proteins from which positive (+) sites were taken, instead of using proteins randomly picked from the Swiss-Prot/TrEMBL database. Thus, both (+) and (-) sites are extracted from the same protein sequences, making our test more strict. Obviously, the (-) sites may contain some false negative hits – these cysteine residues in fact undergo palmitoylation but are not characterized so far. In this regard, the prediction performance of any computational approaches will overestimate the false positive rates. However, without a high-quality gold-standard (-) set, this overestimation is inevitable.

For comparing the prediction performance from NBA-Palm with our previous tool of CSS-Palm [21], both the previously used old data set from CSS-Palm and the new updated data set were used. The detailed information of data description is listed in Table 1.

### Algorithm design and validation

#### Sequence coding

We employed a traditional sliding window strategy to represent a potentially palmitoylated peptide (PPP). Given the window length *n*, a fragment of 2*n *residues centering on palmitoylated site was adopted to represent a PPP. Since there is always C in middle of a PPP, we didn't include the center site into the encoding fragment. We chose an orthogonal binary coding scheme to transform protein sequences into numeric vectors. For example, Glycine was designated as 00000000000000000001, Alanine designated as 00000000000000000010, and so on. The length of final feature vector representing the palmitoylated site is n × 2 ×20. Different values of n varying from 3 to 8 were used to determine the optimized window length.

### The Machine Learning Algorithms

Naïve Bayes is a classification model based on so-called Bayes theorem [22]. Naïve Bayes classifiers assume that the effect of a variable value on a given class is independent of the values of other variables. This assumption is called class conditional independence. It is made to simplify the computation and in this sense is considered to be "Naïve". Given a potential palmitoylation site X, described by its 0–1 feature vector (x_1_, x_2_,..., x_n_) described in above section, we are looking for a class C that maximizes the likelihood: P(X|C)=P(x_1_, x_2_,..., x_n_|C) where C can be "palmitoylation" or "non-palmitoylation". The assumption of class conditional independence allows us to decompose the likelihood to a product of simpler probabilities: P(X|C)=∏i=1nP(xi|C)
 MathType@MTEF@5@5@+=feaafiart1ev1aaatCvAUfKttLearuWrP9MDH5MBPbIqV92AaeXatLxBI9gBaebbnrfifHhDYfgasaacH8akY=wiFfYdH8Gipec8Eeeu0xXdbba9frFj0=OqFfea0dXdd9vqai=hGuQ8kuc9pgc9s8qqaq=dirpe0xb9q8qiLsFr0=vr0=vr0dc8meaabaqaciaacaGaaeqabaqabeGadaaakeaacqWGqbaucqGGOaakcqqGybawcqGG8baFcqWGdbWqcqGGPaqkcqGH9aqpdaqeWbqaaiabdcfaqjabcIcaOiabdIha4naaBaaaleaacqWGPbqAaeqaaOGaeiiFaWNaem4qamKaeiykaKcaleaacqWGPbqAcqGH9aqpcqaIXaqmaeaacqWGUbGBa0Gaey4dIunaaaa@43AE@. Despite of its simple structure and ease of implementation, Naïve Bayes often performs comparatively well with other algorithms, such as SVMs and neural networks.

The support vector machine (SVM) is a new machine learning method, which has been applied for many kinds of pattern recognition problems. The principle of the SVM method is to transform the samples into a high dimension Hilbert space and seek a separating hyperplane in the space. The separating hyperplane, called the optimal separating hyperplane, is chosen in such a way as to maximize its distance from the closest training samples. As a supervised machine learning technology, SVM is well founded theoretically on Statistical Learning Theory [23]. Recently, SVM has been successfully adopted to solve many biological problems, such as predicting protein subcellular locations [32], protein secondary structures [32,33], tumor classification [34] and phosphorylation sites [30,31]. In present work, the feature vector of each potential palmitoylation site was transformed into a higher dimension space through polynomial kernel function.

The RBF network is a kind of multi-layer, feed-forward artificial neural network [24]. An RBF network consists of three layers, namely the input layer, the hidden layer, and the output layer. The input layer broadcasts the coordinates of the input vector to each of the nodes in the hidden layer. Each node in the hidden layer then produces an activation based on the associated radial basis function. Finally, each node in the output layer computes a linear combination of the activations of the hidden nodes. How an RBF network reacts to a given input stimulus is completely determined by the activation functions associated with the hidden nodes and the weights associated with the links between the hidden layer and the output layer. In our model, after feature vectors were fed into input layers, the links between nodes were iteratively updated until convergence. The output layer finally produced the decision of "palmitoylation" or "non-palmitoylation".

### The Jack-Knife validation and n-fold cross-validation

The prediction performances of NBA-Palm were evaluated by the *3*-fold cross-validation, *8*-fold cross-validation and the Jack-Knife validation, for the convenience of comparison with the previous method CSS-Palm. In the Jack-Knife validation, which is also named "leave-one-out" cross-validation, each sample in the dataset is singled out in turn as an independent test sample, and all the remaining samples are used as training data. This process is repeated until every sample is used as test sample one time. In *n*-fold cross validation all the (+) sites and (-) sites were combined and then divided equally into *n *parts, keeping the same distribution of (+) and (-) sites in each part. Then *n*-1 parts were merged into a training data set while the one part left out was taken as a test data set. The average accuracy of *n*-fold cross validation was used to estimate the performance. All models were implemented in the WEKA software package[35].

### Performance measurements

We adopted four frequently considered measurements: accuracy(*Ac*), sensitivity (*Sn*), specificity (*Sp*) and Mathew correlation coefficient (*MCC*). Accuracy(*Ac*) illustrates the correct ratio between both positive (+) and negative (-) data sets, while sensitivity (*Sn*) and specificity (*Sp*) represent the correct prediction ratios of positive (+) and negative data (-) sets respectively. However, when the number of positive data and negative data differ too much from each other, the Mathew correlation coefficient (*MCC*) should be included to evaluate the prediction performance. The value of *MCC *ranges from -1 to 1, and a larger *MCC *value stands for better prediction performance.

Among the data with positive hits by NBA-Palm, the real positives are defined as *true positives *(*TP*), while the others are defined as *false positives *(*FP*). Among the data with negative predictions by NBA-Palm, the real positives are defined as *false negatives *(*FN*), while the others are defined as *true negatives *(*TN*). The performance measurements of sensitivity (Sn), specificity (Sp), accuracy (Ac), and Mathew correlation coefficient (MCC) are all defined as below:

Sn=TPTP+FN
 MathType@MTEF@5@5@+=feaafiart1ev1aaatCvAUfKttLearuWrP9MDH5MBPbIqV92AaeXatLxBI9gBaebbnrfifHhDYfgasaacH8akY=wiFfYdH8Gipec8Eeeu0xXdbba9frFj0=OqFfea0dXdd9vqai=hGuQ8kuc9pgc9s8qqaq=dirpe0xb9q8qiLsFr0=vr0=vr0dc8meaabaqaciaacaGaaeqabaqabeGadaaakeaacqWGtbWucqWGUbGBcqGH9aqpdaWcaaqaaiabdsfaujabdcfaqbqaaiabdsfaujabdcfaqjabgUcaRiabdAeagjabd6eaobaaaaa@3826@, Sp=TNTN+FP
 MathType@MTEF@5@5@+=feaafiart1ev1aaatCvAUfKttLearuWrP9MDH5MBPbIqV92AaeXatLxBI9gBaebbnrfifHhDYfgasaacH8akY=wiFfYdH8Gipec8Eeeu0xXdbba9frFj0=OqFfea0dXdd9vqai=hGuQ8kuc9pgc9s8qqaq=dirpe0xb9q8qiLsFr0=vr0=vr0dc8meaabaqaciaacaGaaeqabaqabeGadaaakeaacqWGtbWucqWGWbaCcqGH9aqpdaWcaaqaaiabdsfaujabd6eaobqaaiabdsfaujabd6eaojabgUcaRiabdAeagjabdcfaqbaaaaa@3826@,

Ac=TP+TNTP+FP+TN+FN
 MathType@MTEF@5@5@+=feaafiart1ev1aaatCvAUfKttLearuWrP9MDH5MBPbIqV92AaeXatLxBI9gBaebbnrfifHhDYfgasaacH8akY=wiFfYdH8Gipec8Eeeu0xXdbba9frFj0=OqFfea0dXdd9vqai=hGuQ8kuc9pgc9s8qqaq=dirpe0xb9q8qiLsFr0=vr0=vr0dc8meaabaqaciaacaGaaeqabaqabeGadaaakeaacqWGbbqqcqWGJbWycqGH9aqpdaWcaaqaaiabdsfaujabdcfaqjabgUcaRiabdsfaujabd6eaobqaaiabdsfaujabdcfaqjabgUcaRiabdAeagjabdcfaqjabgUcaRiabdsfaujabd6eaojabgUcaRiabdAeagjabd6eaobaaaaa@417C@,

MCC=(TP×TN)−(FN×FP)(TP+FN)×(TN+FP)×(TP+FP)×(TN+FN)
 MathType@MTEF@5@5@+=feaafiart1ev1aaatCvAUfKttLearuWrP9MDH5MBPbIqV92AaeXatLxBI9gBaebbnrfifHhDYfgasaacH8akY=wiFfYdH8Gipec8Eeeu0xXdbba9frFj0=OqFfea0dXdd9vqai=hGuQ8kuc9pgc9s8qqaq=dirpe0xb9q8qiLsFr0=vr0=vr0dc8meaabaqaciaacaGaaeqabaqabeGadaaakeaacqWGnbqtcqWGdbWqcqWGdbWqcqGH9aqpdaWcaaqaaiabcIcaOiabdsfaujabdcfaqjabgEna0kabdsfaujabd6eaojabcMcaPiabgkHiTiabcIcaOiabdAeagjabd6eaojabgEna0kabdAeagjabdcfaqjabcMcaPaqaamaakaaabaGaeiikaGIaemivaqLaemiuaaLaey4kaSIaemOrayKaemOta4KaeiykaKIaey41aqRaeiikaGIaemivaqLaemOta4Kaey4kaSIaemOrayKaemiuaaLaeiykaKIaey41aqRaeiikaGIaemivaqLaemiuaaLaey4kaSIaemOrayKaemiuaaLaeiykaKIaey41aqRaeiikaGIaemivaqLaemOta4Kaey4kaSIaemOrayKaemOta4KaeiykaKcaleqaaaaaaaa@65AA@.

### ROC curves

**Figure 2:**
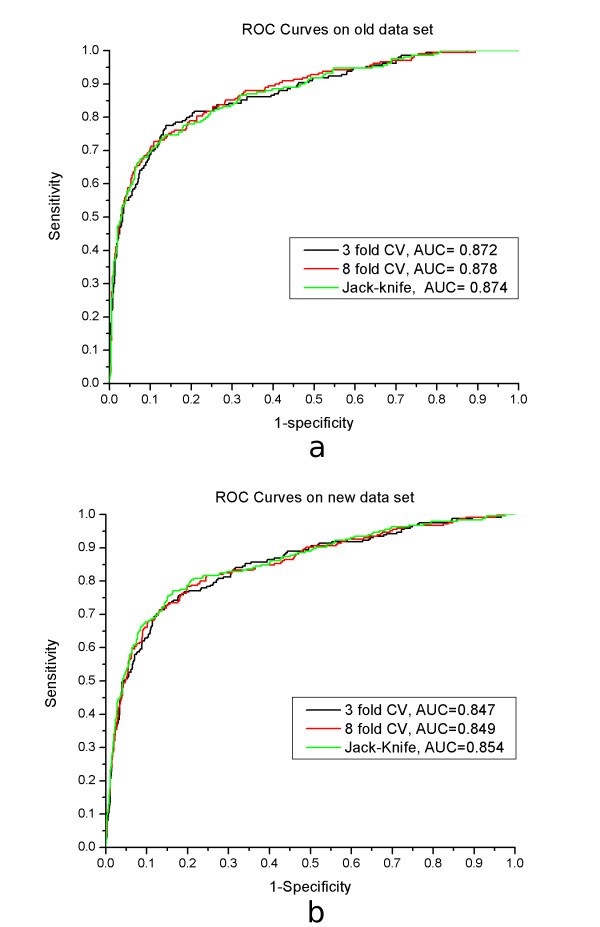
The ROC curves for potential palmitoylated peptides with window length of six. The "3 fold CV" stands for 3 fold cross-validation, the "8 fold CV" for 8 fold cross-validation and the "Jack-Knife" stands for the Jack-Knife validation. The "AUC" stands for Area Under Curve score. (a) ROC curves on old data set; (b) ROC curves on new data set.

The prediction performance of Naïve Bayesian algorithm with window length of six is very similar to that of seven. To compare their performance in detail, ROC curves were used for intuitively visualizing prediction performance (see in Figure 2). ROC curves plot the true positive rate as a function of the false positive rate, which is equal to 1-specificity. The area under the ROC curve (the ROC score) is the average sensitivity over all possible specificity values, which can be used as a measure of prediction performance over different thresholds. ROC curves of random predictors will be around the diagonal line from bottom left to top right with scores of about 0.5, while a perfect predictor will produce a curve along the left and top boundary of the square and will receive a score of one.

## Authors' contributions

YX and HC should be regarded as joint First Authors. YX and HC designed the methodology, carried out the analysis, developed the web service and drafted the manuscript. CJ contributed several insightful opinions and improved manuscript considerably. XY and ZS coordinated the research and finalized the manuscript.
